# Dynamic contrast-enhanced MRI coupled with a subtraction technique is useful for treatment response evaluation of malignant melanoma hepatic metastasis

**DOI:** 10.18632/oncotarget.9567

**Published:** 2016-05-24

**Authors:** Minsu Lee, Song-Ee Baek, Jieun Moon, Yun Ho Roh, Joon Seok Lim, Mi-Suk Park, Myeong-Jin Kim, Honsoul Kim

**Affiliations:** ^1^ Department of Radiology, Yonsei University College of Medicine, Seoul, Republic of Korea; ^2^ Research Institute of Radiological Science, Yonsei University College of Medicine, Seoul, Republic of Korea; ^3^ Biostatistics Collaboration Unit, Yonsei University College of Medicine, Seoul, Republic of Korea

**Keywords:** treatment response, melanoma liver metastasis, magnetic resonance imaging, subtraction technique, mRECIST

## Abstract

**Purpose:**

To determine whether contrast-enhanced MRI including subtraction sequences can predict the treatment response of melanoma liver metastasis.

**Results:**

High precontrast T1 signal intensity (SI) of melanoma lesions obscured detection of enhancement after contrast injection. It was impossible to determine whether or not enhancement occurred in the majority of lesions (85.4%, *n* = 35/41) without including the subtraction technique. Positive enhancement was identified in 14.6% (*n* = 6/41) of patients without subtraction images, but increased to 68.3% (*n* = 28/41) by including subtraction images. Follow-up studies determined lesion progression in 34.1% (*n* = 14/41) of patients. Positive enhancement on the subtraction image (odds ratio = 12.1, *P* = 0.048) and intermediate high T2 SI (odds ratio = 8.16, *P* = 0.040) were significantly associated with higher risk of lesion progression.

**Materials and Methods:**

Patients who underwent MRI for melanoma liver metastases between January 2007 and February 2015 were enrolled. The study analyzed 41 liver metastases in 15 patients [11 male and four female; median age 56 years (range 21–81)] for size, lesion enhancement with and without subtraction images, and T2 SI. Follow-up imaging studies were used to determine treatment response. Data were analyzed with generalized estimating equations.

**Conclusions:**

MRI including the subtraction technique is useful for determining the treatment response of melanoma liver metastases. Lesion contrast enhancement and intermediate high T2 SI increased the risk of lesion progression.

## INTRODUCTION

The incidence of metastatic malignant melanoma is gradually increasing [1–3]. The liver is most frequently involved in metastasis for patients with malignant melanoma. Ocular melanoma metastases show a high predilection for the liver, with frequent occurrence of exclusively hepatic metastases; in turn, metastatic liver disease is the leading cause of death [4–6]. The outcome of patients with liver metastases is generally poor. However, recent advances in melanoma treatment, such as immunotherapy and targeted therapy, provide new therapeutic options and improved treatment outcomes [2, 7].

Early and precise evaluation of treatment response is important for selecting an effective treatment plan, but is often challenging. Most of the methods used for tumor response evaluation, including the response evaluation criteria in solid tumors (RECIST) guideline, are designed to solely evaluate alterations in lesion size [8]. However, the response to anti-cancer therapy is often context-dependent, and it has been suggested that the performance of classical treatment response evaluation systems that primarily focus on anatomical assessments may not be efficient under certain circumstances [9]. For treatment response assessment of melanoma metastases, CT texture analysis or functional evaluation by PET-CT show encouraging results [10, 11]. These results suggest that alternative methods can have a complementary role to classical treatment response guidelines such as RECIST.

Malignant melanoma is hypervascular with high angiogenetic activity [12, 13]. For the modified RECIST (mRECIST) system, which assesses the treatment response of hypervascular hepatocellular carcinoma, only the enhancing region of a mass on the arterial phase of contrast-enhanced images is considered to represent the viable tumor [14, 15]. We hypothesized that the viable tumor concept described in mRECIST could be adopted for treatment response assessment of melanoma liver metastases. Therefore, lesion enhancement characteristics may be more meaningful than lesion anatomical size for treatment response assessment.

The purpose of this study was to determine whether the imaging features of contrast-enhanced dynamic MRI including the subtraction technique can be applied to predict the treatment response of hepatic metastases in patients with malignant melanoma.

## RESULTS

### Study population and treatment response assessment

**Figure 1:**
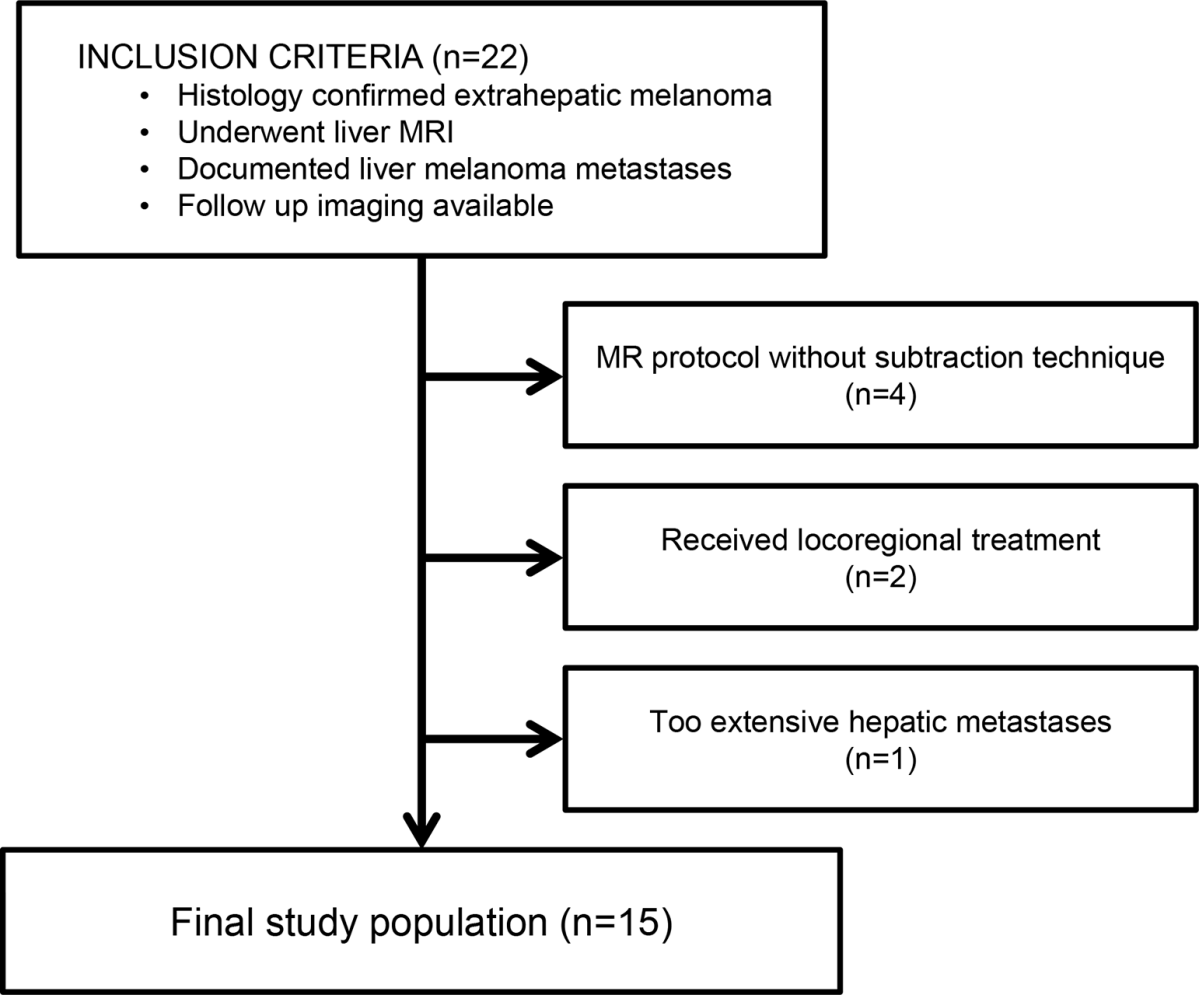
Eligibility criteria of the study population

**Figure 2:**
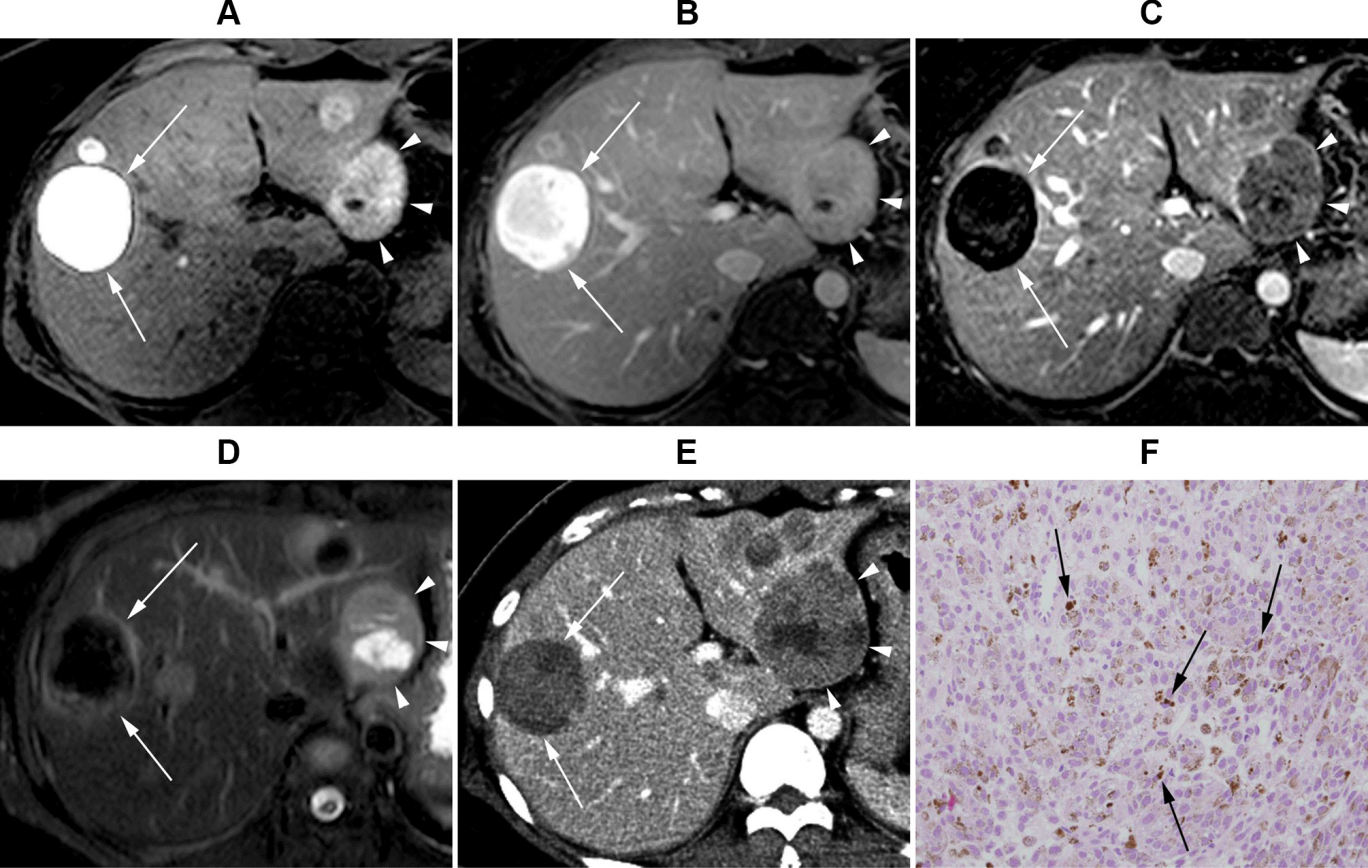
Gadoxeticacid-enhanced liver MRI of a 36-year-old female with uveal melanoma (**A**) Precontrast T1-weighted image. (**B**) Post-contrast portal venous phase T1-weighted image. (**C**) Subtraction image (portal venous phase – precontrast T1 weighted image). (**D**) T2 weighted image. Segment 2 lesion (4.2 cm, arrowheads) displays positive enhancement on the subtraction image and intermediate high T2 signal intensity (SI), whereas segment 8 lesion (4.5 cm, arrows) displays negative enhancement and low T2 SI. (**E**) CT scan image at the same level obtained 2 months later. The segment 2 lesion (arrowheads) size has increased to 6.0 cm, whereas segment 8 lesion (arrows) is still 4.3 cm. (**F**) Histopathological examination [hematoxylin and eosin (H&E) stain ×200] of liver biopsy obtained from the segment 2 lesion revealed tumor cells with abundant intra-cytoplasmic melanin pigments (arrows), consistent with malignant melanoma.

**Figure 3:**
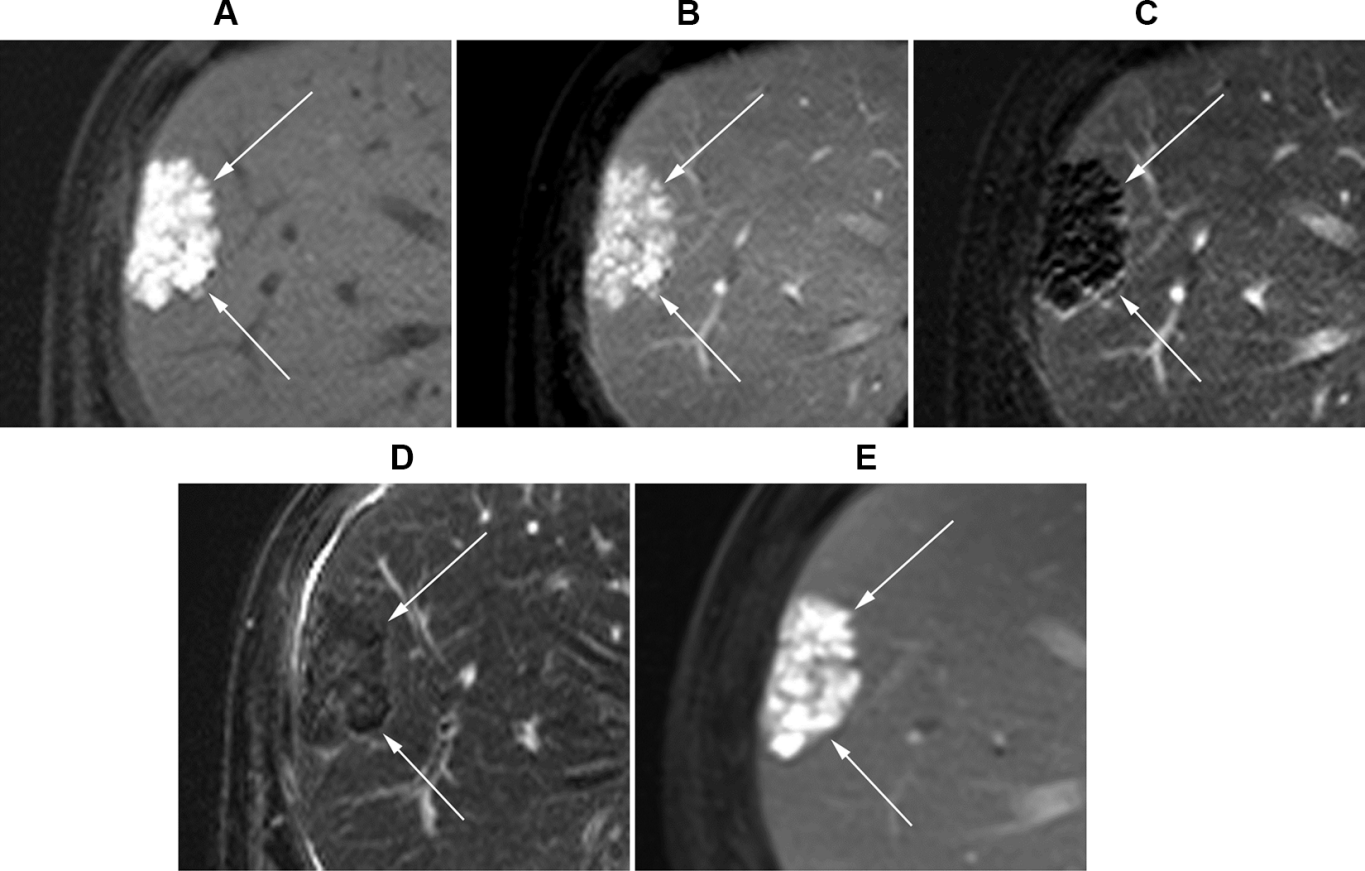
Gadoxeticacid-enhanced liver MR images of a 56-year-old male with uveal melanoma (**A**) Precontrast T1-weighted image. (**B**) Post-contrast portal venous phase T1-weighted image. (**C**) Subtraction image (portal venous phase – precontrast T1-weighted image). (**D**) T2-weighted image. A 4.6 cm metastatic melanoma lesion (arrows) was observed in segment 8, which was graded to show negative enhancement and low T2 SI. (**E**) In follow-up MRI T1-weighted post-contrast-enhanced portal venous phase image, the lesion measured 4.3 cm.

**Figure 4:**
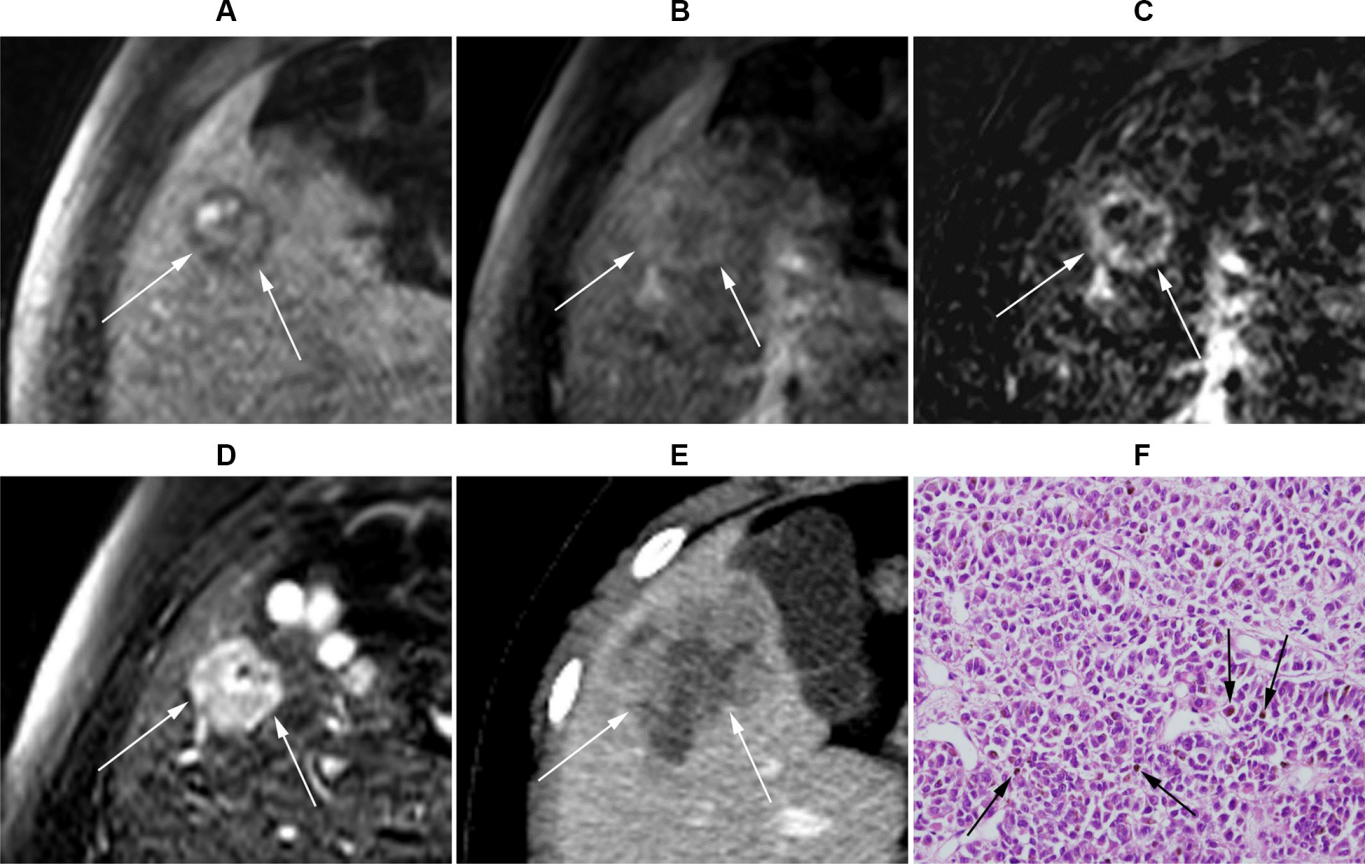
Gadoxetic acid-enhanced MRI of a 21-year-old male with uveal melanoma (**A**) Precontrast T1-weighted MR image. (**B**) Post-contrast-enhanced portal venous phase T1-weighted image. (**C**) Subtraction image (portal venous phase – precontrastT1-weighted image). (**D**) T2-weighted image. A 2.2 cm (arrows) metastasis lesion at segment 5 is observed, which was graded to show positive enhancement and intermediate high T2 SI. (**E**) CT image at the same level obtained 2 months later demonstrated that the lesion size increased to 4.7 cm. (**F**) Histopathological exam (H&E stain ×200) of liver biopsy obtained from the S5 lesion revealed malignant cancer cells with melanin pigments (arrows), consistent with malignant melanoma.

According to the inclusion and exclusion criteria, the study population consisted of 15 patients with 41 metastatic liver lesions (Figure 1, Table 1). The metastatic lesion sizes measured on the precontrast T1-weighted images ranged between 0.7 to 4.7 cm (mean ± standard deviation = 1.78 ± 1.10 cm). On follow-up imaging, all lesions showed a residual lesion with the longest diameter ranging between 0.5 to 6 cm (mean ± standard deviation = 1.98 ± 1.47 cm). Treatment response assessment of each lesion was graded as non-progressive in 65.9% of lesions (*n* = 27/41, Figures 2 and 3) and as progressive in 34.1% of lesions (*n* = 14/41, Figures 2 and 4).

**Table 1:** Demographicsof the study population

Parameter	Data
No. of patients	15
No. of lesions	41
Gender	
No. of men	11 (73.3%)
No. of women	4 (26.7%)
Age in years(median and range)	56 (21–81)
Men	58 (21–73)
Women	61 (39–81)
Mean tumor size (cm) ± standard deviation (range)	1.78 ± 1.10 (0.7–4.7)
Primary Site	
Uvea	13 (86.7%)
Other	2 (13.3%)
Chemotherapy regimen during follow-up	
Dacarbazine	7 (46.7%)
Docetaxel + Carboplatin	2 (13.3%)
Paclitaxel + Carboplatin	1 (6.7%)
Cisplatin + Vincristine + Dacarbazine	1 (6.7%)
Immunotherapy	2 (13.3%)
None	2 (13.3%)

### Imaging characteristics

The presence or absence of enhancement for each lesion was first assessed on the routine dynamic contrast-enhanced images without subtraction images. Specifically, the reviewers compared the precontrast T1- weighted images with the arterial phase and/or portal venous phase images. The reviewers were able to detect positive enhancement in 14.6% (*n* = 6/41) of lesions. However, the reviewers were not able to determine whether or not enhancement existed in 85.4% (*n* = 35/41) of lesions, because the lesions already displayed high signal intensity (SI) on precontrastT1-weighted images (Figures 2A, 3A, and 4A), and the reviewers could not determine whether or not the lesions were further enhanced during the dynamic imaging study (Figures 2B, 3B, and 4B). The interobserver agreement was moderate (Cohen's *κ* value = 0.55; 95% confidence interval, 0.18−0.93).

Next, the reviewers repeated contrast-enhancement assessment with the inclusion of subtraction images. During this assessment, the reviewers determined that 68.3% (*n* = 28/41) of lesions displayed positive enhancement (Figures 2C and 4C), whereas 31.7% (*n* = 13/41) of lesions displayed negative enhancement (Figures 2C and 3C). The interobserver agreement for enhancement based on subtraction sequences was good (Cohen's *κ* value = 0.68; 95% confidence interval, 0.45−0.91) (Table 2).

**Table 2:** Univariate analysis of MR parameters with respect to treatment response

	Number of lesions	Interobserver agreement (Cohen's κ, 95% confidence interval)	Association with treatment response			
			Progression	No progression	*P* value	Odds ratio (95% confidence interval)
Enhancement (analyzed without subtraction image)		0.55 (0.18−0.93)				
Positive	6/41 (14.6%)		66.7% (4/6)	33.3% (2/6)	−	−
Indeterminable	35/41 (85.4%)		−	−		
Enhancement (analyzed with subtraction image)		0.68 (0.45−0.91)			0.048^*^	12.1^*^ (1.02−144.05)
Positive	28/41 (68.3%)		46.4% (13/28)	53.6% (15/28)		
Negative	13/41 (31.7%)		7.7% (1/13)	92.3% (12/13)		
T2 signal intensity		0.85 (0.69−1.0)			0.040^*^	8.16^*^ (1.10–60.67)
Intermediate high SI	23/41 (56.1%)		52.2% (12/23)	47.8% (11/23)		
Hypo-to-isointense SI	18/41 (43.9%)		11.1% (2/18)	88.9% (16/18)		

* Age, gender, and lesion size were adjusted.

Analysis of T2 SI classified 19.5% (*n* = 8/41) of lesions as hypointense (Figures 2D and 3D), 24.4% (*n* = 10/41) of lesions as isointense, and 56.1% (*n* = 23/41) of lesions as intermediate high SI (Figure 4D). None of the lesions displayed high (water) T2 SI. The interobserver agreement on T2 SI was excellent (Cohen's *κ* value = 0.85; 95% confidence interval, 0.69−1.0).

### Imaging parameters associated with treatment response

Analysis of lesion enhancement indicated that 46.4% (*n* = 13/28) of lesions with positive enhancement on subtraction images were progressive lesions, whereas 7.7% (*n* = 1/13) of lesions with negative enhancement were progressive lesions. Analysis with generalized estimating equations indicated that positive enhancement identified on subtraction images was significantly associated with lesion progression [(without adjustment: odds ratio = 10.4;95% confidence interval, 1.14–95.29; *P* = 0.038) and (with adjustment for age, gender, and tumor size:odds ratio = 12.1; 95% confidence interval, 1.02−144.05; *P* = 0.048)] (Table 2).

For intermediate high T2 SI, 52.2% (*n* = 12/23) of lesions were progressive, whereas 11.1% (*n* = 2/18) of hypointense to isointense lesions were progressive. Analysis with generalized estimating equations indicated that intermediate high T2 signal intensity was significantly associated with lesion progression compared with that of isointense to hypointense SI [(without adjustment: odds ratio = 8.73; 95% confidence interval, 1.93−39.38; *P* = 0.005) and (with adjustment for age, gender, and tumor size: odds ratio = 8.16;95% confidence interval, 1.10–60.67; *P* = 0.040)] (Table 2).

## DISCUSSION

Malignant melanoma has a high incidence of metastasis, and prognosis is poor after metastasis has developed [2, 3]. Recent progress in treatment options, such as molecular target agents and immunotherapy, has expanded therapeutic options and the possibility of improved treatment outcome [16]. Therefore, early prediction of therapeutic response is crucial.

Most oncological treatment evaluation systems (such as RECIST) are based on determining changes in tumor size measured on anatomical imaging modalities [8]. However, recent studies suggest that the classical tumor response assessment that is solely based on changes in anatomical tumor size may not always be an efficient predictor of overall survival [10, 17]. Extensive tumor necrosis and/or spontaneous hemorrhage encountered during therapy are well-recognized scenarios that might lead to an increase in tumor size that is irrelevant for the real treatment response [18, 19]. Metastatic melanoma has been reported to frequently develop spontaneous hemorrhage [20–22]. Acute tumor hemorrhage can even be a consequence of rapid tumor response to targeted therapy against melanoma [23], which can result in a paradoxical increase in lesion size.

The mRECIST criteria, which consider only the viable portion of a tumor as actual tumor burden, are widely applied for treatment response assessment of hepatocellular carcinoma. According to this system, only the contrast-enhancing portion of the lesion on arterial phase images is meaningful [14]. Hepatocellular carcinoma and melanoma are both hypervascular tumors [24–27]. Therefore, we speculated that the concept of mRECIST criteria for viable hepatocellular carcinoma tumor assessment also may be valid for assessment of melanoma hepatic metastases, and hypothesized that the tumor enhancement profile on MRI would be associated with treatment response.

Without subtraction images, the assessment of contrast enhancement for melanoma hepatic metastatic tumors on MRI was challenging and was not feasible in most cases. In the majority of lesions (85.4%, *n* = 35/41), it was impossible to determine whether enhancement was present or absent. Melanoma lesions already displayed high T1 SI on precontrast images (Figures 2A, 3A, and 4A), which obscured the analysis of alteration in SI after gadolinium contrast injection (Figures 2B, 3B, and 4B). This T1 hyperintensity is a typical feature of melanoma, which is due to the T1 shortening effect generated by either the melanin pigment or blood products from intratumoral hemorrhages [28, 29].

The subtraction technique has been proven as an effective method to accurately determine lesion enhancement in hepatocellular carcinoma [30–33]. After the inclusion of subtraction images, the reviewers were able to assess the enhancement profile of all lesions: 68.3% (*n* = 28/41) of lesions showed positive enhancement (Figures 2C and 4C), whereas 31.7% (*n* = 13/41) showed negative enhancement (Figures 2C and 3C). Positive enhancement was associated with a higher risk of progression compared with those of lesions without enhancement (odds ratio = 12.1, *P* = 0.048).

Previous literature has reported that the success of subtraction technique lies on the accuracy of coregistration between the enhanced source image set and the precontrast image set [32, 33]. If the precontrast and postcontrast data sets are not obtained during the same breath hold (which is the case of liver MRI used in our study), respiratory misregistration is likely to occur, resulting in pseudo-enhancement artifact. The use of gadoxetic acid also raises an issue, for this contrast agent frequently accompanies acute transient dyspnea resulting in arterial phase image degradation [34, 35] which will accentuate misregistration. Therefore, we assumed that efforts to differentiate true tumor enhancement from pseudo-enhancement would be crucial.

In case of the imaging diagnosis of hepatocellular carcinoma, the presence of not only arterial phase enhancement but also delayed washout is critical and therefore each sequence must be separately analyzed [36, 37]. Meanwhile, we focused to precisely detect any enhancement that might occur within a melanoma metastatic lesion regardless of image phase and decided to analyze both subtraction image sets (arterial phase image set and portal venous phase image set each subtracted by precontrast series) together instead of separately. We believe this to be a simple but efficient way to accurately evaluate the presence or absence of enhancement, because pseudo-enhancement confusing in one image set frequently appears obvious in another image set obtained during a separate breath hold.

The T2 SI imaging parameter was also significantly related to treatment response. Lesions with intermediate highT2 SI had a higher tendency to progress compared with lesions with low-to-isointense SI (odds ratio = 8.16, *P* = 0.040). We believe that this is because T2 intermediate high SI generally represents soft tissue characteristics [38]. Dark T2 SI often represents old hemorrhage [39], which we believe often reflects a chronic, stable nature of the lesion.

Our study has some limitations. First, we did not obtain pathological confirmation for all 41 lesions analyzed. A thorough histological confirmation of stage IV lesions is clinically impossible, and we think that characteristic MRI features, such as precontrast T1 hyperintensity, are highly specific features that justify the imaging diagnoses. However, we acknowledge that our MRI-based approach could have caused selection bias by unintentionally excluding melanoma metastases with less obvious T1 hyperintensity (e.g., amelanotic melanoma). Second, the retrospective nature of this study hindered us from standardizing the follow-up protocol, which could have influenced treatment response assessment. Third, we adopted the concept of viable tumor described in mRECIST criteria, but used the classical concept based on tumor size changes for standard reference of treatment response. We acknowledge that a prospective study will enable more precise survival analysis methods to serve as standard reference for treatment response. Forth, subtraction technique often produces pseudo-enhancement artifact which sometimes closely resemble true enhancement. However, we tried to minimize potential confusion by interpreting multiple subtraction image sets obtained from separate breath holds. Last, the treatment regimen applied to our study population primarily included cytotoxic chemotherapy agents and rarely included molecular target therapy and/or immunotherapy. We recognize the possibility that the response patterns after treatment with these new therapies could differ from those of conventional cytotoxic chemotherapy.

In conclusion, MRI protocols including the subtraction technique are useful for treatment response assessment of melanoma liver metastases. The presence of lesion contrast enhancement and intermediate high T2 SI were significant features associated with a higher risk of lesion progression.

## MATERIALS AND METHODS

### Selection of study population

This retrospective study was approved by our institutional review board with waiver of informed consent. All data were analyzed anonymously. We reviewed our institutional electronic clinical database to collect patients with melanoma who developed hepatic metastasis and underwent liver MRI including the subtraction technique between January 2007 and February 2015. Patients that met all of the following criteria were selected (*n* = 22): (1) patients with histologically confirmed extrahepatic melanoma lesion who underwent liver MRI, (2) documented diagnosis of liver melanoma metastases in the liver biopsy/surgical resection pathology report and/or in the liver MRI report, and (3) follow-up contrast-enhanced liver CT or MRI study performed approximately 1−3 months after the liver MRI. The following exclusion criteria were applied to the selected patients: (1) contrast-enhanced MRI was performed with a protocol that did not include the subtraction technique (*n* = 4), (2) local treatment such as chemoembolization or radiofrequency ablation was performed (*n* = 2), and (3) appropriate lesion selection was impossible due to extensive hepatic metastasis (*n* = 1).

After application of inclusion and exclusion criteria (Figure 1), we obtained a final study population of 15 patients [median age 56 years (range 21–81); 11 males, median age 58 years (range 21–73); four females, median age 61 years (range 39–81)]. In the majority of patients, uveal melanoma was the primary disease site (*n* = 13). Other primary sites were esophagus (*n* = 1) and cutaneous lesion at trunk (*n* = 1).

Six patients had histological evidence of melanoma liver metastasis based on liver biopsy. For the other nine patients who lacked histological evidence, the MRI was reviewed with consensus by two abdominal radiologists (H.K. and S.B., with 4 and 7 years of experience in liver MRI, respectively) to confirm the presence of nodule(s) with bright signal intensity on precontrast T1-weighted images, which is a typical finding for melanoma metastasis [40]. If a patient had multiple liver lesions on MRI, the larger lesions were chosen for analysis by one reviewer (H.K.). However, no more than three lesions were selected from one patient to minimize clustering bias. In total, the study population consisted of 15 patients with 41 metastatic liver lesions.

Eleven patients underwent systemic chemotherapy within the follow-up period with regimens of dacarbazine (*n* = 7), docetaxel and carboplatin (*n* = 2), paclitaxel and carboplatin (*n* = 1), or cisplatin, vinblastine, and dacarbazine (*n* = 1). Two patients received immunotherapy with dendritic cell vaccination and nivolumab injection, respectively. Two patients were untreated due to poor general condition (Table 1).

### MR imaging

MR images were obtained using a 3-T MRI scanner (Magnetom Tim Trio, Siemens Healthcare, Erlangen, Germany; or Achieva, Philips Healthcare, Best, the Netherlands) and a previously published protocol [41]. The following sequences were captured: a breath-hold transverse T1-weighted in- and out-of-phase two-dimensional (2D) gradient-echo (GRE) sequence (TR/in phase TE, 150/2.4 msec; out-of-phase TE, 1.2 msec; flip angle, 65°; FOV, 32– 38 × 25–29 cm; matrix, 256 × 256; section thickness, 6 mm; slice spacing, 1.2 mm; number of slices, 30); a breath-hold transverse 3D GRE (TR/TE, 2.5/0.9 msec; flip angle, 13°; FOV, 32–36 × 25–36 cm; matrix, 320 × 224; section thickness, 2 mm; no gap; acquisition time, 23 sec) with fat suppression technique; and a T2-weighted turbo spin echo (TR/TE, 466/96; FOV, 32–36 × 25–29 cm; matrix, 256 × 192; section thickness, 5 mm; slice spacing, 1 mm). The protocol underwent detailed adjustments according to individual demands whenever necessary.

Dynamic MRI was performed by injecting an intravenous bolus of contrast agent through a 20-gauge intravenous catheter placed into a peripheral vein, followed by a 20-mL saline flush at the rate of 2 mL/ sec. Contrast agents included 0.025 mmol/kg body weight gadoxetic acid (*n* = 13; Primovist; Bayer Schering Pharma, Berlin, Germany) and 0.1 mmol/kg body weight gadobenic acid (*n* = 2; Multihance; Bracco Imaging, Milan, Italy). A breath-hold transverse 3D GRE sequence with fat suppression was obtained before and after contrast agent injection. Arterial phase was acquired at 2 or 3 seconds after peak aortic enhancement according to a bolus-tracking method, followed by portal venous, hepatic venous, and transitional phase images with intervals of 30 to 40 seconds each. Hepatobiliary phase images were obtained at 15 to 20 minutes after injection of contrast agent when gadoxetic acid was used. Subtraction images were obtained by automatically subtracting the unenhanced series from arterial and portal venous phase images.

### Image analysis

All MRI studies were archived in digital imaging and communications in medicine (DICOM) format on picture archiving and communication system (PACS) workstations (Centricity, GE Healthcare, Waukesha, WI, USA). The initial MR images of the candidates were reviewed by two abdominal radiologists (H.K. and S.B., with 4 and 7 years of experience in liver MRI, respectively) who were aware that melanoma liver metastasis was suspected, but were blinded to all other clinical information. A reviewer (H.K.) measured the longest lesion diameters directly on the PACS. Analyses of other imaging parameters were conducted in two separate sessions. Initially, the two readers independently assessed the MRI images. Subsequently, the results with discrepancies were reassessed based on consensus agreement of the reviewers.

The analyzed imaging parameters included lesion enhancement and T2 signal intensity. The presence or absence of enhancement was evaluated in two steps. (1) First, each reader visually compared routine arterial and/or portal phase images with the precontrast T1-weighted images. At this point, the images reconstructed with the subtraction technique were not provided. (2) Next, the subtraction images obtained by subtracting the precontrast images from the arterial and portal phase images were provided to the reviewers to reassess the enhancement pattern. The T2 signal intensity of each lesion was graded as low SI (lesion appeared darker when compared with the adjacent liver parenchyma), isointense SI (similar brightness of lesion when compared with the adjacent liver parenchyma), or intermediate high SI (lesion appeared mild to moderately brighter when compared with the adjacent liver parenchyma) based on comparison with the SI of liver parenchyma.

### Follow-Up imaging and treatment response assessment

The follow-up period ranged from 38 to 86 days (mean ± standard deviation = 55.1 ± 13.3 days). Follow-up imaging studies were based on CT (*n* = 9) or MRI (*n* = 6). For follow-up CT examinations, images were obtained with a 64-channel scanner (Sensation 64, Siemens Healthcare, Erlangen, Germany). Patients were intravenously injected by power injector via the antecubital vein at a dose of 1−2 mL/kg nonionic contrast material (Omnipaque 300; GE Healthcare) during 30 seconds. A bolus-tracking technique was employed to obtain the portal venous phase 55 seconds after the Hounsfield unit value of the abdominal aorta had increased by 100 Hounsfield units compared with that of baseline. Imaging parameters were as follows: 0.5 second rotation time; 120 kV; reference mAs, 240 mAs with automated tube current modulation; beam collimation, 0.6 mm; beam pitch, 1; and 3 mm slice thickness. Follow-up MRI examinations were performed with the same imaging parameters as the initial MRI.

The longest diameter of each lesion on follow-up study was measured by an abdominal radiologist (H.K.). The treatment response of each lesion was graded as either progressive or non-progressive. We defined a lesion as progressive if the longest diameter on follow-up imaging increased by at least 20%. All lesions that were not determined as progressive were classified as non-progressive lesions [adopted from [8]].

### Statistical analysis

Statistical analysis was performed using SAS version 9.3 (SAS Institute, Cary, NC, USA). During statistical analysis, the T2 SI grades were collapsed into binary values to enhance convenience and clarity of analysis (low SI and isointense SI lesions versus intermediate high SI lesions). The Cohen's kappa value was calculated for each imaging parameter to estimate interobserver agreement. Interobserver agreement value (*κ*) was classified as follows: 0−0.20, poor agreement; 0.21−0.40, fair agreement; 0.41−0.60, moderate agreement; 0.61−0.80, good agreement; and 0.81−1.00, excellent agreement. To evaluate the effects of data involving lesions from the same patient, we performed univariable/multivariable analysis by adopting generalized estimating equations to determine the statistical relevance of MRI findings (enhancement on subtraction sequences and T2 signal intensity) for treatment responses. However, T2 SI was omitted in multivariable analysis because multicollinearity existed between enhancement on subtraction images and T2 signal intensity. A result was considered significant for differences with *P* value < 0.05.
